# Integrative Multi-Omic Signatures of Longevity and Healthy Aging

**DOI:** 10.1093/geroni/igaf122.2881

**Published:** 2025-12-31

**Authors:** Lance Pflieger, Kengo Watanabe, Theinmozhi Arulraj, Max Robinson, Oliver Fiehn, Robert Moritz, Jodi Lapidus, Noa Rappaport

**Affiliations:** Phenome Health, Seattle, Washington, United States; Tokushima University Graduate School of Biomedical Sciences, Tokushima, Tokushima, Japan; Institute for Systems Biology, Seattle, Washington, United States; Institute for Systems Biology, Seattle, Washington, United States; University of California, Davis, Davis, California, United States; Institute for Systems Biology, Seattle, Washington, United States; Oregon Health & Science University, Portland, Oregon, United States; Institute for Systems Biology, Buck Institute, Phenome Health, Seattle, Washington, United States

## Abstract

There is a growing interest in serum-based biomarkers that reveal the mechanisms of healthy aging. The Longevity Consortium generated untargeted mass spectrometry-based proteomic and metabolomic datasets from serum samples across four cohorts; the Osteoporotic Fractures in Men (MrOS) Study, the Study of Osteoporotic Fractures (SOF), the Health, Aging, and Body Composition (Health ABC) Study, and the Cardiovascular Health Study (CHS) totaling 3380 total participants with a 1:3 case-cohort design. This dataset offers a unique opportunity to identify a robust prospective signature of Longevity, defined as survival to the 98th percentile based on sex- and birth cohort-specific survival distributions. We applied multiblock sparse partial least squares discriminant analysis and systems biology approaches to integrate and construct multi-omic signatures predictive of longevity. From approximately 5000 metabolites and 500 proteins, we identified biomarkers associated with coagulation and complement and cholesterol transport. Interestingly, the cholesterol transport signature (apolipoproteins) was only enriched in females, suggesting sex-specific mechanisms of longevity. Furthermore, we validate these findings by comparing omics data from human centenarian and mouse longevity studies that also capture molecular signatures induced by life-extending interventions. This study underscores the power of integrative systems biology methods in characterizing the heterogeneity of molecular aging phenotypes, ultimately enabling the development of robust longevity signatures. The identified biomarker signatures have potential implications for personalized interventions aimed at promoting healthy aging and mitigating age-related diseases.

